# HELENA project: Driving innovation in high energy density Li-metal halide solid-state batteries for electric vehicles and aircrafts

**DOI:** 10.1016/j.csbj.2025.03.013

**Published:** 2025-03-19

**Authors:** Pedro López-Aranguren, Pierre Lannelongue, Javier Carrasco, Andrey Golov, Adrian Robles-Fernandez, Elena Gonzalo, Artur Tron, Thomas Marchandier, Anna-Katharina Hatz, Reza Fathi, Qi Xu, Diana Chaykina, Cerun Alex Varkey, Claudio Gavagnin, Matteo Kaminski, Alaa Almousli, Gebrekidan Gebresilassie Eshetu, Niloofar Hamzelui, Fabian Diaz, Emanuel Drude, Sara Abada, Nicolas Guy, Akshayan Sudharshan, Massimo Brunetti, Stefan Beschnitt

**Affiliations:** a Center for Cooperative Research on Alternative Energies (CIC energiGUNE), Basque Research and Technology Alliance (BRTA), Parque Tecnológico de Álava, Vitoria-Gasteiz, Spain; b IKERBASQUE, Basque Foundation for Science, Plaza Euskadi 5, Bilbao 48009, Spain; c AIT Austrian Institute of Technology GmbH, Center for Low-Emission Transport, Battery Technologies, Vienna, Austria; d Saint-Gobain Research Paris, Aubervilliers 93303, France; e Umicore, 31 rue du Marais, Brussels 1000, Belgium; f Lionvolt, High Tech Campus, 31, Eindhoven 5656 AE, Netherlands; g TNO-Holst Centre, High Tech Campus 31, Eindhoven 5656 AE, the Netherlands; h Fraunhofer Gesellschaft zur Foerderung der Angewandten Forschung ev, Hansastrasse 27c, Munchen 80686, Germany; i Institut für Partikeltechnik Technische Universität Braunschweig Volkmaroder, Str. 5, Braunschweig 38104, Germany; j Customcells Holding Gmbh, Fraunhoferstrasse 1 b, Itzehoe 25524, Germany; k Institute of Power Electronics and Electrical Drives (ISEA), RWTH Aachen University, Campus Boulevard 89, Aachen 52074, Germany; l Mimitech Gmbh, Preusweg 98, Aachen 52074, Germany; m LFP Energies Nouvelles, Avenue de Bois Preau 1 & 4,, Rueil Malmaison 92500, France; n Pipistrel Vertical Solutions, Vipavska cesta 2, Ajdovscina 5270, Slovenia; o Leonardo, Piazza Monte Grappa 4, Roma 00195, Italy; p FEV Europe Gmbh, Neuenhofstrasse 181, Aachen 52078, Germany

**Keywords:** Halides, Solid-state batteries, Electric vehicles, Electric aviation, Climate neutrality, Battery roadmap

## Abstract

The development of sustainable, high-efficiency and reliable batteries is critical to support climate neutrality goals, particularly in the electromobility sector. The European-funded HELENA project (Halide Solid State Batteries for Electric Vehicles and Aircraft) addresses these challenges by focusing on the manufacturing of next-generation lithium-metal solid-state batteries. The project tackles key hurdles in raw material sourcing, battery production, sustainability and cost-effectiveness. A multidisciplinary partnership brings the innovation from the lab to the industrial scale, covering the whole value chain, including industrial material producers, R&D centres, battery manufacturers, and automotive and aerospace end-users. The cell design features a high-voltage nickel-rich cathode coupled with a high-energy lithium metal anode and a lithium-ion superionic halide solid electrolyte. This configuration enhances energy and power density, thus being suitable for electric vehicles and aircrafts. HELENA aims to reshape the solid-state battery landscape by advancing technology readiness levels through the manufacturing of 10 Ah pre-industrial prototypes. This report highlights mid-way results of the project, discussing the major advancements in battery specifications and safety standards from end-users, processing of materials into battery component, battery modelling and recycling strategies to ensure long-term sustainability.

## Graphical Abstract

**Figure:**
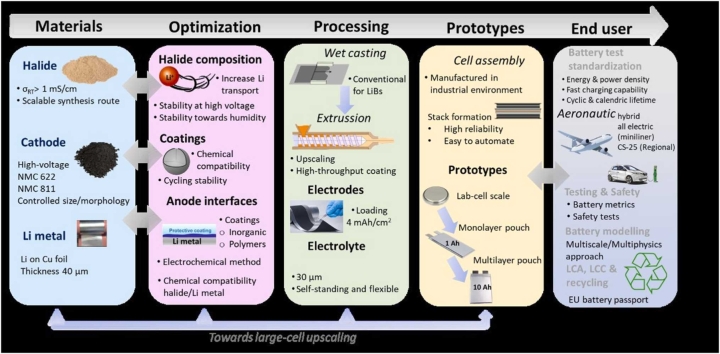

## 1 Introduction

Reducing greenhouse gas emissions is critical in the global effort to combat climate change, and the electrification of main transport sectors has emerged as a cornerstone strategy. Transportation contributes significantly to worldwide emissions, particularly from road vehicles and aviation, offering a substantial opportunity to mitigate global warming. In this transition, advancements in battery technology play a relevant role, not only in meeting current energy demand but also in enabling innovations that can shape a sustainable future progress. The global battery industry is experiencing rapid growth, driven by increasing demand from the transport sector. By 2030, advancements in battery technology are expected to lower global emissions by up to 30 %, accelerating the shift towards a sustainable, low-carbon future.

The HELENA project (Halide Solid State Batteries for Electric Vehicles and Aircraft [1]) is part of a broad European initiative of collaborative projects [2], [3], [4], [5], [6], [7] aimed at boosting next generation battery technology to meet the immediate needs of industry. The project focuses on solid-state batteries (SSBs) designed to deliver enhanced energy and power density for both electric vehicles and aircrafts. The proposed technology addresses the limitations of conventional lithium-ion batteries by providing scalable, cost-effective, and safer energy storage solutions. Driven by innovative materials, the project targets advancements in long-distance travel, fast-charging capabilities, and improved safety standards, critical milestones for the future of electromobility. In aviation, which contributes approximately 3.5 % of the total of CO_2_ emissions, the transition to electric propulsion faces unique challenges due to the high energy density required for flights. Therefore, the development of solid-state batteries for small-scale commercial aviation, represents a first step towards broader adoption of electric aircraft.

This project is contributing to the dynamic industry landscape by introducing novel materials, such as Li-ion superionic halide electrolytes which, in combination with high-capacity Ni-rich cathodes and high-energy Li metal anode, hold promise to advance SSB technology. The project focuses on key performance indicators on materials properties, manufacturing processes, cost-efficiency, and recycling strategies, all of which are essential for bringing these technologies to market.

HELENA is on track to advance the technology to higher technology readiness levels, with efforts underway to scale up to pre-industrial prototypes of 10Ah. This report presents the project's mid-term achievements, including a detailed discussion of the guidelines derived from automotive and aviation battery specification requirements. Key results from the initial stages of cell manufacturing are highlighted, with validated components at cell level representing the starting point to build pouch cell prototypes, and supported by battery modelling. Additionally, we address critical sustainability aspects, emphasizing the development of new recycling strategies to minimize the environmental footprint of battery production.

## 2 Project description

### 2.1 Objectives of the project

**Fig. 1:**
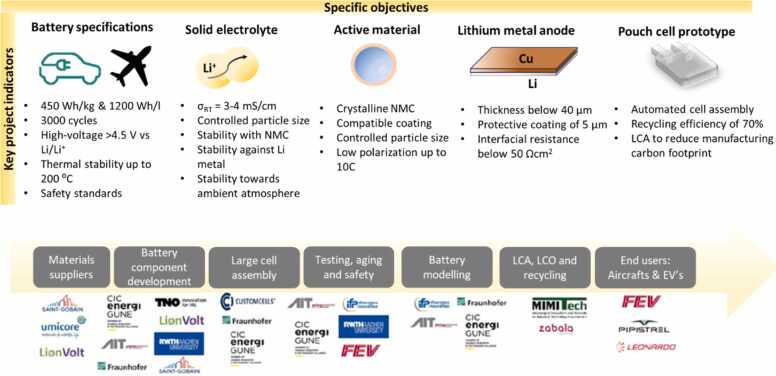
Overview of HELENA’s roadmap for battery development, highlighting key project indicators, specific objectives and collaborative network value chain.

The HELENA partnership is developing a Li-metal halide-based solid-state cell, with an overall focus on material innovation and a sustainable and cost-competitive cell manufacturing process. This is accomplished through a step-by-step development with specific objectives supported by key indicators (Fig. 1), starting from the definition of battery specifications by end-users, sourcing of key materials, such as the solid electrolyte, active material and lithium metal anode, manufacturing battery components and the final assembly of 10 Ah pre-industrial prototype cells.

To ensure the successful achievement of these ambitious objectives, ***CIC energiGUNE*** [8] coordinates the project with a multidisciplinary consortium spanning the entire battery value chain, bringing together industrial material producers **(*****Saint-Gobain Recherche*** [9], ***Umicore*** [10]), start-up companies, R&D centres and universities **(*****Lionvolt*** [11], ***Dutch Organization for Applied Scientific Research (TNO)*** [12]***, Fraunhofer Institute for Surface Engineering and Thin Films*** [13] ***IFP Energies Nouvelles*** [14]***, Austrian Institute of Technology*** [15], ***MIMI tech*** [16], ***RWTH Aachen University*** [17]) battery manufacturers (***CustomCells*** [18]) and automotive (***FEV Software and Testing solutions*** [19]**)** and aerospace end-users ***(Pipistrel*** [20], ***Leonardo*** [21]). ***Zabala*** [22] is leading the dissemination and exploitation tasks, ensuring the project's long-term impact on society.

### 2.2 Cell concept and methodology

**Fig. 2:**
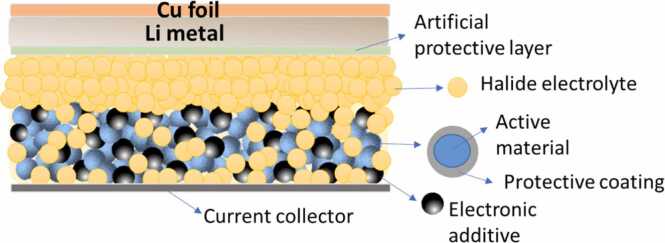
HELENA’s cross-sectional schematic cell concept highlighting key components and their arrangement.

HELENA’s SSB concept (Fig. 2) relies on a halide electrolyte, a high-voltage layered-oxide active material and a Li metal anode with a protective layer. The solid electrolyte is the main material that sets the battery apart from LiBs and is the enabler of high-energy and -power density device expected to have improved safety features.

Over the last decades, solid electrolytes such as sulfides [23], [24] and oxides [25], [26] have been heavily investigated for their commercial application in SSBs [27], [28], [29]. Table S1 provides a comparison with existing LiBs and new generation of solid-state battery benchmarks[30]. However, there are technological challenges still hampering the industrial deployment of these chemistries. For instance, sulfide electrolytes face safety issues with the formation of H_2_S in contact with humidity, requiring a controlled dry atmosphere for their handling. Oxides, in contrast, require a high-temperature sintering process that increases cell manufacturing costs[31]. In this scenario, halide-based Li-ion conductors have recently become appealing candidates by bringing the best features from sulfides and oxides. They exhibit a high conductivity over 2 mS cm^–1^, they can be cold-pressed into thin (∼30 µm) low density membranes (2.4 g cm^–^^3^), and they are chemically safe [32]. Within the HELENA project, halide materials are first optimized at the R&D level to match the battery specifications of the project, and then industrially upscaled for the cell manufacturing.

High-voltage (up to 4.5 V vs Li/Li^+^) layered LiNi_x_Mn_y_Co_z_O_2_ (x + y + z = 1) (NMC) cathodes, such as NMC622 and NMC811, are strategic materials offering long cycle life, high power, and energy density, making them ideal for electromobility applications. NMC622 exhibits a moderate specific energy (165 mAh g^–1^) with a low internal resistance and a high structural stability enhanced by the presence of Mn. NMC811, despite the lower structural stability, offers a higher capacity (210 mAh g^–1^) and improved rate capability due to the higher Ni content, electronic conductivity, and ionic diffusivity. Additionally, NMC811 reduces battery costs by minimizing the Co content. In this project, the NMC composition is specifically designed and optimized for integration with halide-based chemistries. The nickel content is systematically increased from NMC622 to NMC811 to achieve higher operating voltages, thereby enhancing the energy density of the material.

At the anode side, HELENA focuses on the use of high-capacity Li metal. This anode material brings a major advantage to SSBs with respect to energy density [33]. Solid electrolytes enable the use of high-capacity lithium-metal anodes, as their mechanical strength may prevent dendrite growth. However, the successful integration of the anode with a solid electrolyte requires mitigating the reactivity between both materials and ensuring a low interfacial resistance [34], [35]. Therefore, the development of protective interfacial layers is established as key strategy to address challenges of the Li metal anode.

**Fig. 3:**
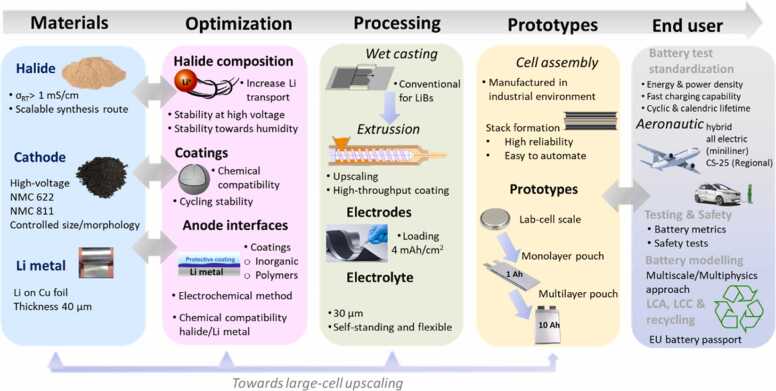
Outline of the battery development in HELENA, from material selection and optimisation to processing, prototyping and final application, emphasizing scalability and performance evaluation for end-user readiness.

A methodology for coordinating and planning R&D activities has been defined for the realistic assessment of integrating materials into SSB prototypes. Fig. 3 provides a comprehensive overview of the process involved in the development and upscaling of battery technology, moving from materials selection through optimization, processing, prototyping and ultimately to end-user applications.

HELENA project’s methodology also includes battery modelling, which is a transversal activity to accelerate the improvement of materials and battery features. Computational methods support the experimental research, reducing costs for materials, personnel, and equipment. Within the HELENA project, these methods are used to analyse interfaces, ionic and electron transport, mechanical behaviour, and thermal properties from the atomic level to complete battery packs. The final goal is to implement a multiscale-multiphysics battery model, offering model-based guidance to optimize cell and electrode design and anticipate full cell behaviour and lifetime.

The overall methodology of HELENA can be broken down in three main phases:

**Phase I**. Development, optimization, and upscaling of new materials.

In this phase, the main effort is focused on developing advanced materials for battery components, particularly halide solid electrolytes, cathodes, and anodes. This phase will ensure that the supplied materials meet the necessary specifications for high-performance batteries. Main objectives of this phase include ***optimization*** (i.e., fine-tuning material properties for better performance, safety, and compatibility with manufacturing processes), ***scaling up*** (i.e., upscaling halide and active materials to meet the requirements of battery development stages, from lab testing to large prototype fabrication), and ***assessment*** (i.e., finalizing suitable processes for upscaling coating and other key steps to ensure industrial applicability and cost-effectiveness).

The target for the halide electrolytes in this phase of the project is to develop different compositions and morphologies, while applying low-cost and scalable methods. As key target features, halide density must be around 2 g cm^–3^ to achieve high energy densities, and a high ionic conductivity above 2 mS cm^–1^ to ensure high-power applications. Besides, the powders must be compatible with extrusion processing for ease of manufacturing and integration into prototype pouch cells.

On the cathode side, the project begins with NMC622 and aims to integrate NMC811 to reach energy density targets for the final cells. The optimization of these materials aims to address challenges like surface reactivity and structural integrity. For instance, developing advanced coatings is a strategy to improve compatibility with halides to maintain low irreversible capacity and high C-rate performance.

On the anode side, using untreated thin lithium metal foils (below 40 μm in thickness) with a halide solid electrolyte can form a poor Li-conducting solid electrolyte interphase (SEI), leading to low-performing batteries with a short cycle life. HELENA aims to develop and upscale advanced interfacial layers by identifying compatible chemistries and optimizing their properties for high Li conductivity, dendrite suppression, and stable SEI formation. Inorganic and organic coatings are envisaged as protective layers, applying methods such as spatial atomic layer deposition and a roll-to-roll electrochemical pre-formation process from liquid electrolytes. The final goal is to process lithium via evaporation to achieve thin (< 40 μm) anode, followed by the interfacial protection (Fig. 3), considering cost and material circularity.

**Phase II**. Halide based solid-state battery development.

**Fig. 4:**
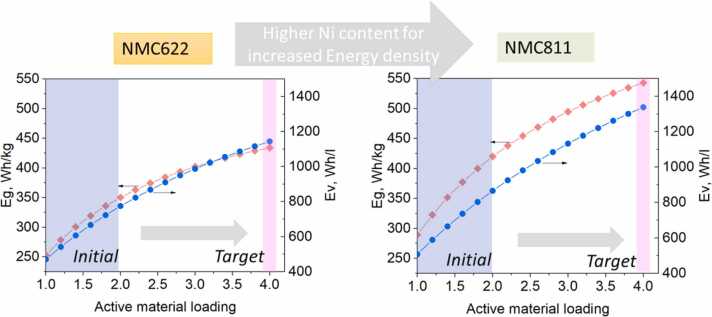
Projected energy density improvements within HELENA as active material shifts from NMC622 to NMC811, with emphasis on increasing active material loading as key strategy on the transition.

In this phase, the project focuses on developing SSBs at lab- and pre-prototype scale with optimized materials from Phase I. First, the materials from *phase I* are processed into a format that can be used in pouch cells. (Fig. 3). Halide solid electrolyte powders are processed as thin membrane separators, targeting a thickness of 30 μm. Composite positive electrodes are manufactured by combining the active material with the halide electrolyte and conductive carbon additive, with an organic binder to form dense layers. Besides using a thin Li metal solid electrolyte, achieving the desired gravimetric and volumetric energy densities involves a careful balance of the active material loading on the cathode formulation. The electrode loadings are therefore gradually increased from 1 to 2 up to 4 mAh cm^–2^ to reach gravimetric and volumetric energy density targets (Fig. 4), making necessary adjustments to the formulation and processing techniques.

The overall goal is to produce laminates with optimized formulations that exhibit low resistivity, high-rate capability, and long-term cycling. Detailed interfacial characterization from lab-scale cells to larger prototypes is crucial for optimizing the technology to achieve high performances, particularly by reducing the cell resistance for faster charging. Upscaling involves adapting lab-scale electrode/electrolyte coating and cell assembly processes for pre-industrial prototypes. To produce a sufficient amount of cathode/electrolyte composites, mixing techniques will be scaled up to 50 L batch sizes, with the ultimate goal of developing a continuous, less solvent-intensive extrusion method (Fig. 3). This method is preferred due to its lower cost and environmental impact. With these laminates, multilayer pouch cell prototypes of 1–10 Ah will be manufactured in industrial-scale environments on a fully automatic production line to evaluate performance and safety indicators.

**Phase III**. Testing, optimization and validation.

Herein pouch cell devices of 1–10 Ah are tested for validation of cell performance and safety levels against the unified set of requirements for EV and aviation. The data generated in this phase also validates the numerical battery model that guides material developers and cell manufacturers to predict full cell performance and lifetime, and address challenges from the atomistic to the macro-level. In addition, this phase includes a recycling strategy, defining sustainability and cost targets for materials and final prototypes. This strategy integrates life cycle assessment and cost (LCA, LCC) along with an experimental series to determine the best process parameters for optimizing recycling efficiencies, distribution coefficients, and recoverable resources.

## 3 Results and outcomes of the project

### 3.1 Consolidated requirements and testing protocols for automotive and aeronautic sectors

A comprehensive set of technical requirements and specifications for battery cells in aeronautics and automotive applications has been defined, with a strong emphasis on safety. While automotive and aerospace sectors share some overall battery requirements, the key difference lies in the significant weight penalty in aerospace applications, which has a high impact on the performance and the final design of the battery pack. In addition, the safety and reliability factors are significant, which might influence the overall battery requirements as well as the final design of the aircraft. Despite substantial advancements in energy and power capabilities over the past three decades, driven by extensive research from academia and battery manufacturers, enhancing battery performance for long-distance travel and fast charging, while ensuring high safety standards remains a critical priority. Table 1 provides a detailed summary of the high-level requirement specifications defined within this project. For automotive applications, the estimated minimum specific gravimetric energy density is 400 Wh kg^–1^ while the ideal volumetric energy density is above 850 Wh L^–1^. The peak specific power is mostly derived from the acceleration requirements for automotive applications, (i.e.; the minimum has to be higher than 1200 W kg^–1^ and the peak power density is preferably higher than 2000 W L^–1^). Fast-charging stations are installed on the road network to facilitate quicker charging time compared to the regular charging, therefore, the cells must be capable of charging from 10 % to 80 % SOC (State of Charge) in less than 20 minutes. This corresponds to a maximum charge rate of 3 C at 25 ℃ at beginning of life (BOL). Since the battery is operated in the SOC range between 20 % and 90 %, the power should not be derated in this window. The targeted lifetime for automotive applications is given as the equivalent full cycle of 750 at 70 % SOH (State of Health). For the SOC window between 20 % and 80 %, it is 1000 cycles at 90 % SOH.

**Table 1:** List of consolidated SSB cell requirements for EVs and aircraft.

**Specification**	**Aircraft**	**EVs**
***E***_***g***_	450 Wh kg^–1^	450 Wh kg^–1^*(C/3 discharge rate, T = 25°C, BOL)*
***E***_***v***_	1200 Wh L^–1^	
***Max. cont. disch. current (T = 25°C)***	5 C (before thermal cut off is reached)	2 C
***Equivalent cont. disch. power***	10 C *(SOC above 20 %, T = 25°C −50°C)*	5 C
***Peak current (T = 25°C)***		
***Equivalent peak disch. power***	4 C *(SOC=0 %−60 %, T = 25°C −50°C)*	3 C *(SOC=20 %−80 %, T = 25°C, BOL)*
***Max. peak charge current***		
***Cycle life***	3000 cycles *(DoD > 90 %, T = 25°C −50°C, 1 C charge-1C discharge,*$C_{\mathrm{Eol}}=0.8C_{\mathrm{BoL}}$*;*$R_{\mathrm{EoL}}=1.3R_{\mathrm{BoL}})$	750 Cycles *(Equivalent full cycles, T = 25°C, C/2 or C/3 discharge rate, SOH 70 %)*
***Operating temp. range for full perf.***	+ 25 to + 45 °C	−20 to > 50 °C
***Operating temp. range with reduced power***	−10 to + 25 ºC + 45 to + 60 °C	−30 to + 60 °C
***Survivability***	−45 °C + 85°C	−45 °C + 80 °C *(EUCAR hazard level 0)*
***Self discharge***	< 1 % per month for SOC= 20 % 80 %; T = 45°C	< 2 % SOC at 100 % SOC (open circuit) at 45 °C per month
***Certification requirements***	UN38.3	
***Costs***	< 75 €/kWh	

Aeronautic requirements are divided into Battery Electric Aircraft (BEA) and Hybrid Electric Aircraft (HEA) categories, each having their own needs on the performance and safety requirements. Table 1 combines the requirements for the next generation of small electric commuter BEA and HEA aircraft into a single set of requirements. While automotive batteries typically deliver moderate, sustained power for daily driving, aircraft batteries emphasize higher power output for takeoff and within a wider temperature range, reflecting the more strict performance and safety requirements in aerospace.

**Fig. 5:**
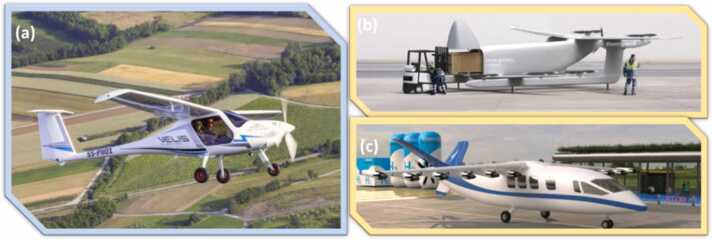
(a) Pipistrel Velis Electro type certified all electric aircraft (b) A concept art of the NUUVA V300 hybrid electric aircraft, (c) A concept art of the UNIFIER19 hybrid electric aircraft.

The Pipistrel Velis Electro (Fig. 5), world’s first electric powered aircraft to receive a Type Certificate, can fly for 50 minutes (plus VFR reserve). To date, the Pipistrel Velis Electro is the only type-certified all-electric aircraft mainly capable of flight trainings. Although a flight time of 50 minutes is sufficient for typical flight school utilisation with 2 passengers, it is not enough for thin-haul and short-haul aircraft, which cover passengers from 19 up to 80 with typical range of 500 – 1000 km. Nevertheless, the Pipistrel Velis Electro pioneers the reference to estimate the cell requirements for the next generation of BEA and HEA.

### 3.2 Materials optimization and component manufacturing

Inorganic solid electrolytes for SSBs must meet numerous stringent requirements, including high ionic conductivity, wide electrochemical stability, and compatibility with lithium metal. Beyond these electrochemical properties, considerations such as particle size distribution, scalable synthesis methods, stability towards ambient atmosphere, and feasibility for membrane manufacturing are crucial. Achieving these attributes while minimizing costs is one of the key industrial challenges.

To date, only oxides and sulfides have shown potential for large-scale production. However, halides have recently gained attention due to their promising features as solid electrolytes and perspective for their manufacturing at large scale. Li₃MX₆ halide compounds exhibit hexagonal or crystal structures with a high ionic conductivity due to weak Coulombic interactions and a high Li/M ratio, which together lower migration barriers and create extensive Li-ion diffusion networks. One of the earliest demonstrations of this potential came from Panasonic in 2018, measuring a conductivity of 1 mS cm⁻¹ for LYC and LYB [36]. Following studies have explored a variety of metal cations (e.g., Y, In, Sc), halide anions (F, Cl, Br), and both isovalent and aliovalent substitutions to optimize cation/anion sizes, introduce vacancies, and enhance charge carriers [32]. These modifications have a strong influence on lithium-ion pathways and overall conductivity.

Halide electrolytes are appealing materials for industrial large-scale manufacturing due to their versatile synthesis methods. Halides can be prepared via ball milling, co-melting, or wet chemical methods [17] for producing a large variety of compositions [37], [38]. Wet chemistry, in particular, is suitable for upscaling because it simplifies processing, decreasing energy consumption and production costs while keeping high ionic conductivity of the electrolyte [16]. Although halide electrolyte compositions may include expensive metals, this can be mitigated through careful material selection and substitution with aliovalent metals. At industrial scale, the choice of metals and halides is constrained by cost, solubility, and safety concerns, limiting the use of elements like scandium, indium, zirconium, and fluorine. For example, Li₂ZrCl₆ has shown high humidity tolerance and high ionic conductivity [18], [19], [20]. Compared to rare metals like indium, zirconium is cheaper and more abundant, thus reducing overall costs. Furthermore, the increase in production volumes is expected to drive prices down on the market [15].

**Fig. 6:**
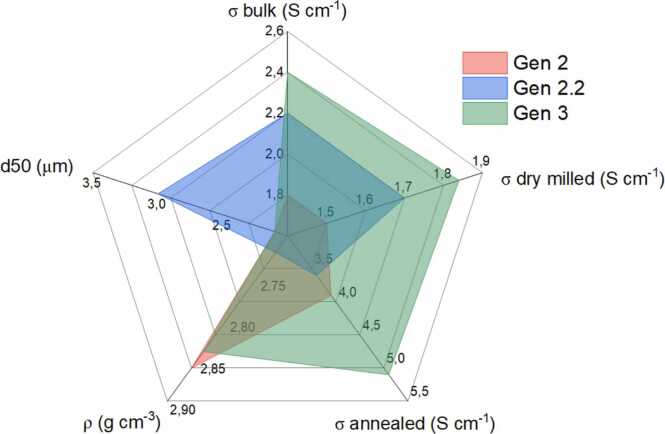
Properties of halide generations developed in HELENA.

In HELENA, halide compounds, Li_3_YBr_6_ (Gen 1) Li_3_YBr_2.1_Cl_3.9_ (Gen 2), Li_3_YBrCl_5_ (Gen 2.2) and Li_3_Y_1-x_M_x_Br_2_Cl_4_ (Gen 3) were developed, with each generation showing enhanced properties (Fig. 6). Ionic conductivity and particle size were identified as key parameters to ensure optimal battery performance. Improvements in ionic conductivity (σ_bulk_) were achieved through crystal chemistry by adjusting the halide stoichiometry in Gen 1 and Gen 2 and further enhanced in Gen 3 by Yttrium substitution. In solid-state batteries, particle size plays a critical role. Unlike liquid electrolytes, solid particles do not fill available space naturally, making porosity, tortuosity, and contact surface crucial factors. On the one hand, for electrolyte separators, maximum conductivity is achieved with minimal porosity and small, uniformly sized particles. On the other hand, for positive electrode formulations, the balance between ionic and electronic conductivity, active material quantity, and particle contact is key to ensure redox processes. Research has shown that the use of smaller electrolyte particles enhances the cycling capacity of the cell by improving conductivity, reducing porosity and improving particle-particle contact [38], [39]. In this project, the systematic optimization of dry milling parameters allowed the production of particles below 3 µm with narrow size distribution (d50), balancing the benefits of reduced particle size with potential impacts on crystallinity and ionic conductivity (σ_dry milled_). Indeed, as observed in Fig. 6, the size reduction also increases the number of interfaces, decreasing crystallinity and consequently the ionic conductivity. Annealing of the halide under temperature allows for a further increase of the ionic conductivity (σ_annealed_ = 5.1 mS cm^−1^), which places Gen 3 among the highest solid ionic conductors (Fig. 6).

**Fig. 7:**
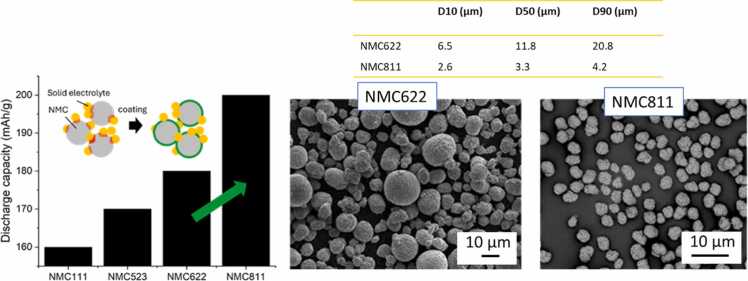
Optimization of discharge capacity in NMC active materials with increasing Ni content. SEM micrographs of NMC622 and NMC811 particles with reduction in particle size.

Regarding the positive active material, HELENA is focused on developing high-voltage cathode composites with high capacity and active material loading in order to reach energy densities above 400 Wh kg^–1^ (Fig. 4). Recent research on layered-oxide NMC active materials has increasingly aimed at boosting the energy density by raising the Ni content in "Ni-rich" compositions. Beginning with NMC622 (60 % Ni, 20 % Mn, 20 % Co), which offers a specific capacity of 170 mAh g^–1^, efforts have advanced to NMC811 (80 % Ni, 10 % Mn, 10 % Co), achieving capacities of up to 210 mAh g^–1^. Ni-Mn-Co materials offer enhanced electrochemical performance and stability while ensuring recyclability. Umicore has tailored NMC622 and NMC811 (Fig. 7) for halide-based SSBs by doping for improved structural stability, and by adjusting the morphology and particle size for optimal cycling performance. Additionally, to ensure an electrochemically stable interface between the halide solid electrolyte and NMC, a surface coating may be introduced [40]. Even though chloride-based halide SEs like Li_3_InCl_6_ exhibit favourable theoretical electrochemical stability windows, the decomposition of halide solid electrolytes in contact with NMC materials, especially with high Ni-content, was observed in the literature [41], [42]. It is crucial to prevent the reactivity between these materials, since this leads to an irreversible loss in capacity, limiting the cycle life of the cell and limiting rate performance due to the formation of thick resistive layers containing decomposition products. A theoretical study suggests coatings for NMC with respect to phase stability, electrochemical stability, and chemical stability with chloride-based halide SEs [43]. In HELENA, a screening of cathode active material grades with different particle properties and surface coatings in lab-scale cells was used to identify the best combination of materials. In a second phase, Umicore increased the production of these materials to kg scale, to provide them for the upscaling into larger multilayer pouch cells.

Fig. 7 shows the increase in discharge capacity with an increase of Ni content in NMC materials as well as the application of coatings to increase the interface stability between halide solid electrolyte and NMC. On the right, the scanning electron microscopy (SEM) images of polycrystalline NMC622 and NMC811 grades fine-tuned for Halide chemistry, meeting the energy density and cost goals are shown.

In the following steps of positive electrode manufacturing, the successful integration of the active material in a composite cathode relies in the careful optimization of the formulation and processing of the materials, which will be discussed in the following section [44].

### 3.3 Towards cell manufacturing

**Fig. 8:**
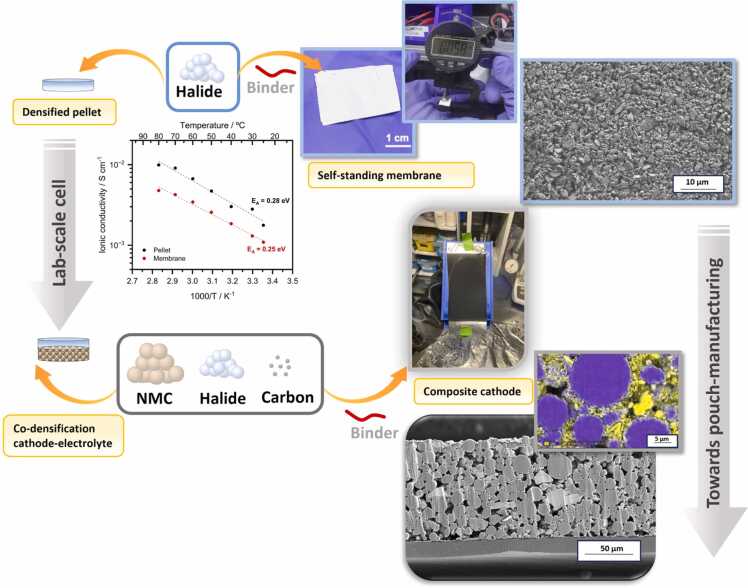
Manufactured components for pouch cells devices. Halide powder and composite cathode can be densified to prepare lab-scale cells. The processing of the powders with a polymer binder allows to obtain self-standing membranes and composite cathode laminates as battery components for pouch-cell manufacturing.

The primary goal of the HELENA project in battery manufacturing is to scale up halide-based solid batteries from 1 mAh lab-scale cells to 10 Ah multilayer pouch cell prototype devices. At the lab-scale, coin cells are usually assembled from densified halide pellets and cathode composite powders [45] (Fig. 8). For larger prototype devices, the processing of these components needs to be adapted, aiming to obtain a thin SE layer and a sufficient active area. The key challenge lies in manufacturing a thin, but mechanically robust halide electrolyte membrane, and a cathode laminate, while maintaining an ionic and electronic conductivity at the highest active material loading possible. By achieving a targeted thickness for the halide membranes of around 30 μm (comparable to the separator in conventional liquid Li-ion batteries), we can lower the cell's ohmic resistance, thereby improving performance [46]. At the same time, the separator must keep key properties such as high ionic conductivity, a high relative density, and sufficient flexibility for handling [47].

In the HELENA project, the electrochemical performance of materials at lab-scale is underway and has shown promising perspectives [48], [49]. For the upscaling of the technology from lab to pouch cell format, solid electrolyte self-standing membranes have been prepared from the mixture of halide powder and a binder in an organic solvent [50], paying special attention to the choice of these components to ensure their chemical compatibility. Furthermore, balancing the binder content is about trading-off high ionic conductivity against high enough binder content to ensure mechanical stability of the membrane, but low enough to ensure a high ionic conductivity (Fig. 8). Finally, the ratio between solid and liquid must be equilibrated to obtain an optimal viscosity of the slurry. A too high or low viscosity has an impact on the quality of the membrane by introducing some defects such as particles agglomerations or by inducing a large amount of pores [51]. After a screening of several materials and ratios, 60 μm thick and flexible membranes have been prepared for cell assembly (Fig. 8). The membranes exhibit a high ionic conductivity of 1 mS cm^−^^1^ at RT. Decreasing the thickness down to the target of 30 μm will be part of the next steps of the membrane fabrication process optimization.

The most common method to fabricate composite cathode laminates is via casting of a slurry on an aluminum current collector. However, the cathode formulation for solid-state batteries differs from conventional liquid batteries due to the nature of the solid electrolyte. In conventional batteries, the cathode layer is composed of active material mixed with a binder and a conductive additive which, respectively, ensure the mechanical integrity and the electronic conductivity of the electrode [52]. The liquid electrolyte can fill the pores of the cathode, ensuring the transport kinetics to obtain high capacity under high current density. To provide the necessary ionic transport in solid-state batteries, the electrolyte needs to be integrated as part of the cathode formulation [53]. As such, the mass ratio between active material and solid electrolyte will play an important role on the performance of the battery, as a very high content of active material will lead to high capacities at low rates, but poor rate capability. On the opposite extreme, too much solid electrolyte will limit the capacity. An extensive study of the formulation performed within HELENA has led to an optimized composition of 75–80 wt% of active material and 19–24 wt% of solid electrolyte, along with 1 wt% of conductive additive.

Producing high energy density batteries requires the manufacturing of cathodes with high loading of active material. The fabrication of high-loading cathode laminates from a slurry casting method has several challenges because the thicker the coated electrode, the more likely it is to have defects such as cracks and delamination [54]. Crack formation generally occurs during the drying step and it is a result of the lack of adhesion of the composite cathode materials to the current collector. Similar to the solid electrolyte membrane, the quantity of binder and solid content is of importance for the mechanical integrity of the cathode. In this project, cathode laminates of high capacity and mass loading up to 3 mAh cm^−2^ and 17 mg cm^−2^ have been prepared with binder contents of 1 wt% and a total solid content of 75 wt%. Fig. 8 illustrates a cathode composite laminate and a cross-section SEM image prepared by ion-milling. The observed porosity negatively impacts ion transport due to the lack of contact between the active material and electrolyte. However, the porosity can be decreased by applying uniaxial pressure at 390 MPa for a few minutes.

Volume changes of the active material are expected during cycling, which can lead to a loss of contact between the active material and solid electrolyte particles. This can be observed as a reduced ionic conductivity, or higher resistance in the cell. To keep this phenomenon negligible, a constant pressure is applied during cycling, which is commonly reported for solid-state batteries [55].

The interface between the halide solid electrolyte and the anode is also a key aspect of the battery, since (electro)chemical instability and the formation of SEI layers can inhibit the performance of the cell [56]. In general, halide-based solid electrolytes face stability issues primarily at the interface with anodes such as Li metal and are typically regarded as being stable towards oxidation with some cathodes [43], [57], [58]. This is due to the reduction of the metal cation in the solid electrolyte upon contact with Li, forming an SEI layer composed of Li-based salts (e.g., LiF, LiCl, LiBr) and metallic species (Y metal in the case of Y-based electrolytes) [59], [60], [61]. Due to the mixed ionic/electronic conductivity (MIEC) of this interphase, this decomposition reaction may be continuous or non-self-limiting. To mitigate this, a protective layer can be applied to either the Li metal anode or the halide solid electrolyte membrane [62], [63] which would prevent the continuous decomposition of the anode and electrolyte, stabilising the interface. This approach needs to be verified and implemented at several levels: (1) choosing the right chemistry for this artificial SEI, (2) applying the layer and testing its potential for interface passivation at the lab-scale, and (3) adapting the approach for up-scaled processing. The aim of this project is to develop organic or inorganic interface layers that lead to an interface resistance below 50 Ω-cm^2^. To achieve this, we focus on scalable techniques, one of which is spatial atomic layer deposition (sALD) performed at TNO, which can be used to deposit ultra-thin (on the order of nanometres) layers on complex materials. As well, since this deposition is done at atmospheric pressure, the throughput is increased substantially compared to conventional ALD, with several companies in the market that have roll-to-roll spatial ALD systems available.

**Fig. 9:**
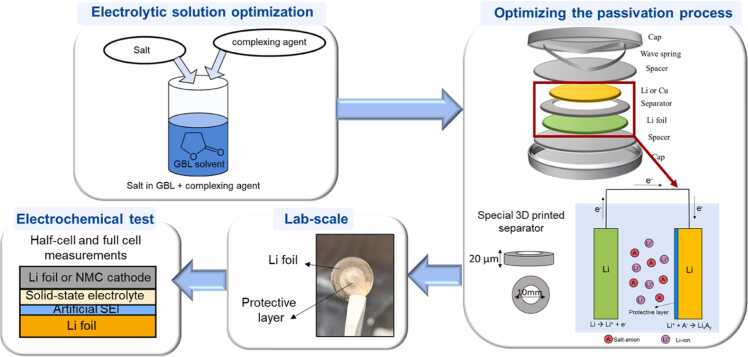
Process of optimization and formation of protective layer on Li surface in lab-scale.

The electrochemical modification of the Li metal surface is also a suitable approach. Here, surface and interface modification of the Li metal was conducted using electrolytic bath containing an innovative salt anion dissolved in gamma-butyrolactone. Due to the low solubility of the salt, a complexing agent was added and improved the ionic conductivity of the electrolytic bath, thus, improving the electrochemically driven modification of the Li metal surface. To avoid the direct contact between the two Li metal electrodes in the symmetric cell, a special separator was used. A homogeneous coating (Fig. 9) was obtained and is currently being tested against the halide solid electrolytes.

### 3.4 Multiscale-multiphysics modelling framework

**Fig. 10:**
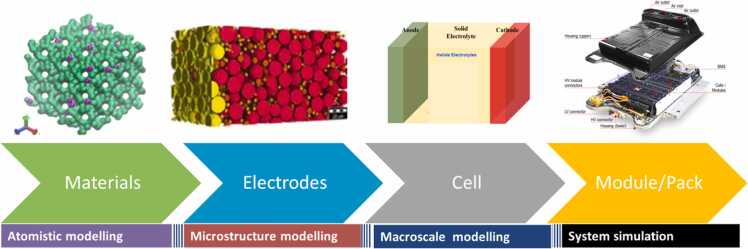
Hierarchical modelling approach for HELENA: from atomistic modelling of materials to system-level simulations of modules and packs, encompassing microstructural, macroscale, and electrode-level analysis.

Multiscale-multiphysics modelling is a computational approach that integrates simulations across different scales, from atomic to macroscopic levels, to better understand and optimize materials and devices (Fig. 10). In battery development, this method helps improve materials properties, enhance battery performance, and predict long-term behavior [64].

At the atomic level, models focus on lithium-ion diffusion through electrolytes and their interactions with electrodes. Moving to the microstructural level, models simulate mechanical stress and strain within battery components, which arise from volume changes during lithiation and delithiation. This scale also considers particle-particle contact, porosity, and tortuosity, which affect both ionic and electronic conductivity. Growth of dendrites and crack propagatio at the mesoscale may lead to battery failures [65], [66]. At the macroscale, models integrate all these effects to simulate battery performance, aging, and thermal behavior under operational conditions. Finally, system simulations evaluate the average behavior of cells in realistic battery pack use cases within automotive and aeronautic applications.

The HELENA project employs multiscale-multiphysics modelling to provide a structured framework for optimizing battery design. This helps manufacturers enhance battery efficiency, durability, and safety by predicting how materials and cells behave over time. The approach is particularly valuable for SSBs, where new materials such as halide-based solid electrolytes are being explored.

#### 3.4.1 Atomistic modelling

The atomistic modelling approach involves simulating materials at the atomic level to predict fundamental properties like ionic diffusion, mechanical behavior, and electrochemical stability. A key technique used is density functional theory (DFT), conducted by CICE, which helps predict materials properties, especially for solid electrolytes interacting with lithium metal anodes [48].

Our research findings indicate that lithium mobility is highly dependent on the specific crystallographic sites occupied by the ions. Additionally, simulations show that the SEI is highly reactive, with a reaction front continuously propagating from lithium into the bulk of the electrolyte, which suggests instability of the SEI. In contrast, our studies on the interfaces between solid electrolytes and high-voltage cathodes reveal controlled reactivity, ensuring stability without affecting the bulk material. In particular, we performed simulations of the interfaces between the solid electrolyte and high-voltage cathode materials such as NMC611 and NMC811 [67], finding that the reaction is limited to the interface region and does not impact on the bulk structure of the electrolyte or cathode; therefore, the interface is expected to be stable. These findings provide critical insights into stability, and reactivity at the SEI with cathode and anode materials, which are essential for developing next-generation halide-based SSBs with enhanced performance.

#### 3.4.2 Microstructural modelling

The microscale modelling conducted by Fraunhofer IST focused on simulating the behavior of composite cathodes using the discrete element method (DEM) to analyze how applied pressure and volume changes affect battery performance. Specifically, two case studies were considered: one where increasing calendaring pressure was applied to a representative volume element (RVE), and another where constant stack pressure was applied, followed by volume changes due to lithium intercalation.

Our findings show that the contact area between the active material and the solid electrolyte increases with applied pressure and volume growth, but even at high pressure, the total surface contact is never achieved, influencing ionic transport and overall battery performance. Additionally, the DEM simulations reveal that higher pressures reduce the porosity of the cathode, which is crucial for optimizing both ionic and electronic conductivity.

Furthermore, the results highlight the significance of inter-particle contact areas and mechanical stress distribution within the composite cathode. In particular, the simulations demonstrated that the lithium intercalation process leads to changes in volume that affect the mechanical stability of the electrode, causing compression and increased internal stresses. These insights are critical for designing more robust SSBs, as they show how mechanical degradation can be mitigated by controlling pressure and particle contact areas during the fabrication process. The simulation outputs are being used to inform the optimization of HELENA’s SSBs, particularly in enhancing their mechanical durability and improving their performance under cyclic operation.

#### 3.4.3 Mesoscale/macroscale modelling

Macroscale modelling focuses on simulating entire battery cells under real-world conditions. It integrates findings from atomic and microstructural levels to study performance, aging, and safety. The implemented macroscale models were developed by IFPEN using a single particle model with electrolyte (SPMe) approach which is based on the reduction of the Doyle-Fuller-Newman model [68], adapting it to address the specific challenges posed by SSBs. The SPMe model integrates electrochemical reactions, lithium-ion transport, stress effects, and the evolution of the interface between the solid electrolyte and the Li anode, a critical issue for the safety and longevity of solid-state batteries. This model is able to upscale and assess cell performance, aging and safety from coin cell to large cell format.

At the mesoscale level, the phase-field method [61] which is well-suited for capturing complex electrochemical and mechanical interactions, is used to predict the conditions under which lithium dendrites initiate and propagate, potentially leading to short circuits. Simulations indicated that dendrite formation is driven by overpotential and mechanical stresses at the interface. The model effectively captures the anisotropic growth of lithium metal and the formation of dendrites, emphasizing how mechanical stresses can influence dendrite growth in relation to confinement, that tends to inhibit dendrite formation when increased. Furthermore, by coupling mechanical deformation with electrochemical processes, the simulations demonstrate that stress concentrations within the electrolyte could lead to cracking and void formation in the solid electrolyte, thereby promoting dendrite growth when the electrolyte material is compromised. The simulations also suggest that defects or inhomogeneities in the electrolyte structure could serve as nucleation points for dendrites. These insights are essential for designing more robust solid electrolytes and developing strategies to mitigate dendrite formation, thereby improving the safety and durability of SSBs.

#### 3.4.4 Battery pack system simulation

The main objective of the system modelling approach is to evaluate the average behavior of cells in realistic battery pack use cases within automotive and aeronautic applications. The battery pack system simulation will be adapted and extended to meet the requirements of the specified automotive and aeronautic use cases, resulting in concept designs for aeronautic batteries. The system simulation will be enhanced with cell-scale models, specifically by integrating the electrochemical model either directly through FMU (functional mock-up unit) or as a fast electrical and thermal equivalent circuit cell model, which is derived from testing the electrochemical model in a virtual environment. A sensitivity analysis will be performed to assess how the uncertainties at the lower scale (cell level) affect the battery performance in realistic applications.

Overall, the multiscale-multiphysics modelling framework developed within the project offers a comprehensive approach to optimizing the next generation of batteries, particularly SSBs. By integrating insights from atomic to macroscopic scales, the project enhances understanding and prediction of the behavior of key components such as solid electrolytes, active material cathodes, and lithium metal anodes. This integrated modelling approach has not only improved the understanding of material interactions within SSBs but also provided guidance for optimizing battery design for improved performance, safety, and longevity. The project's collaborative work between experimentalists and theorists have yielded actionable insights, with the potential for significant advancements in battery technology.

### 3.5 Recycling targets, strategy and safety requirements

HELENA focuses on developing effective recycling strategies for its SSBs, which present unique challenges due to their complex composition. These challenges primarily arise from the presence of materials like metallic lithium, halide electrolyte, and NMC active material, making standard recycling approaches for conventional LiBs insufficient. The project’s recycling approach emphasizes safety, material recovery, and compliance with the evolving regulatory framework.

**Fig. 11:**
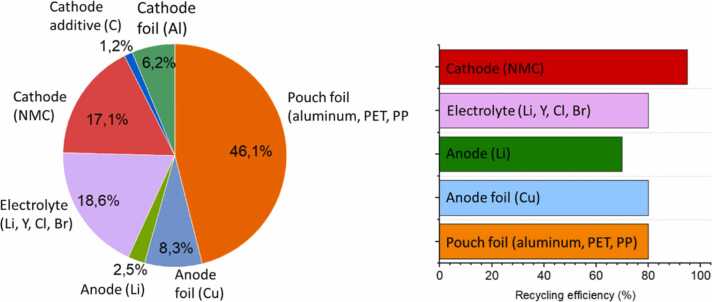
HELENA’s material matrix and expected extraction yield targets.

The recycling targets set by the project aim for an overall recycling efficiency of 75 %, with specific goals for different materials (Fig. 11). These targets align with upcoming European Union regulations, which set a 65 % total recycling rate for LiBs by 2025, increasing to 70 % by 2030. For key metals such as cobalt, copper, nickel, and lead, the EU requires a 90 % recycling rate by 2027, while lithium has a 50 % target.

The project’s approach goes beyond these requirements for some materials, notably targeting an 80 % recycling efficiency for lithium recovered from halide electrolytes. This ambitious target is achievable due to lithium compounds' selective solubility in polar solvents. Other materials, such as copper and NMC cathodes, also have high recovery targets based on previous research [27], [28], [29], [32], and existing industrial-scale recycling concepts.

**Fig. 12:**
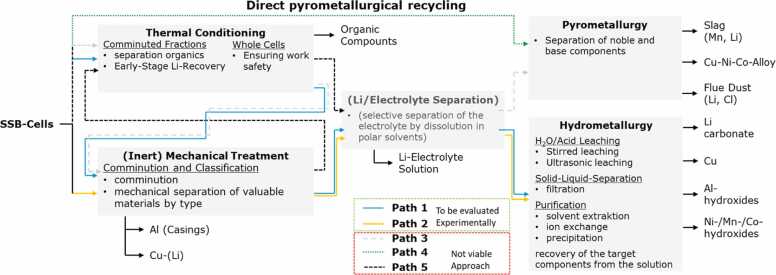
Schematic process flow for HELENA’s cell chemistry, including thermal, mechanical, and hydrometallurgical methods.

While some of the cell’s materials resemble those found in conventional LIBs, including copper current collectors, NMC cathodes, and graphitic additives, the inclusion of halide electrolytes and lithium metal introduces new recycling challenges. Lithium, being highly reactive with a low melting point, poses risks during both thermal and mechanical treatments. Additionally, the electrolyte’s chemical properties may complicate recycling processes. Thermochemical Simulations indicate that halide electrolytes, like the 3YCl_3_-LiCl system, could break down into various compounds when exposed to heat or water. This phenomenon demands special precautions to control emissions and hazardous byproducts. To address these challenges, HELENA’s recycling strategy integrates thermal conditioning, mechanical treatment, and hydrometallurgical processing. These techniques, while aligned with industry standards, are tailored to HELENA’s specific battery composition. The main recycling pathways considered in this project are outlined in Fig. 12.

The first recycling route (path 1 in Fig. 12) provides a comprehensive material recovery process by integrating thermal conditioning, mechanical treatment, and hydrometallurgical processing. The goal of thermal conditioning is to stabilize reactive components like lithium and modify the chemical structure of the electrolyte, enhancing later separation processes. For this battery chemistry, precise temperature control is crucial to prevent the decomposition of halide electrolyte constituents like LiCl and YCl_3_, which could release hazardous byproducts such as hydrochloric acid if overheated. While thermal conditioning is essential for streamlining subsequent treatments, it must be carefully controlled due to the sensitivity of these materials (see Figs. S1,S2, S3 and S4).

After thermal conditioning, mechanical treatment physically separates components such as copper foils, aluminum, and the valuable black mass, which contains key metals like nickel, cobalt, manganese, and lithium. This step is carried out under an inert atmosphere, typically using argon gas to prevent lithium from reacting with moisture or oxygen. A major challenge during mechanical treatment is the high reactivity of lithium metal, which must be kept isolated from oxygen to prevent the formation of undesired compounds like lithium hydroxide or lithium oxide. Once mechanical separation is complete, the black mass undergoes further processing, primarily via hydrometallurgical methods.

Hydrometallurgical processing in the project focuses on selectively recovering metals through acid leaching, typically using sulfuric or hydrochloric acid. This process dissolves metals into a solution, enabling their selective extraction or precipitation. A major challenge in halide-based SSB recycling is the potential contamination of the leachate with halide electrolytes. To mitigate this, precise control over the solubility of lithium compounds is necessary to ensure high recovery rates of lithium, nickel, cobalt, and manganese while minimizing material losses.

An alternative recycling route (path 2 Fig. 12) skips the thermal conditioning step, instead relying on mechanical treatment followed directly by hydrometallurgical processing. This "cold route" is particularly suitable for batteries containing temperature-sensitive halide electrolytes, which may degrade when exposed to heat. In this process, the batteries are shredded at ambient temperatures under an inert atmosphere, similar to the first recycling route. After mechanical separation, acid leaching is used for metal recovery. While this approach eliminates the thermal risks associated with heating halide electrolytes, it presents challenges in isolating lithium metal, which may remain mixed in the black mass, potentially hindering the recovery of other metals during hydrometallurgical processing.

Another recycling route (paths 3 and 4 in Fig. 12) involves direct pyrometallurgical processing, in which battery materials are subjected to extreme heat (1300°C–1600°C). This technique recovers metals like nickel, cobalt, and copper as slag or alloyed forms. Although pyrometallurgy is widely applied in conventional battery recycling, it is less suited for HELENA’s SSBs due to the presence of lithium metal and halide electrolytes. Besides the risk of halide electrolyte decomposition, lithium’s low melting point (180°C) means it would vaporize or become lost in slag or dust, leading to significant material losses.

The final recycling route (path 5 in Fig. 12) combines mechanical treatment with controlled thermal conditioning, offering a balanced approach that minimizes the limitations of individual processes. In this method, batteries first undergo mechanical separation under inert conditions before being subjected to low-temperature thermal conditioning. This step modifies the structure of the remaining materials while preventing the breakdown of halide electrolytes or excessive lithium loss. By keeping the temperature moderate, the process avoids the release of volatile compounds while preparing materials for further hydrometallurgical recovery. This combined strategy requires precise temperature control and process optimization to ensure HELENA’s battery components are adequately stabilized before further processing.

The efficiency of these recycling approaches depends on continued research and refinement, particularly in optimizing halide electrolyte management and minimizing lithium losses. As the project advances, ongoing experimentation will be crucial to scaling these recycling methods to industrial levels while ensuring compliance with strict environmental regulations.

The proposed recycling pathways have been well-defined in terms of process efficiency, material recovery, and safety considerations. However, economic feasibility and industrial scalability are not within the scope of this project. That said, the well-developed pyrometallurgical and hydrometallurgical industrial technologies currently used for end-of-life LIBs recycling remain the reference point for this research approach. Rather than designing a completely new and standalone recycling route—an approach unlikely to achieve feasibility in the future—this research aims to bridge the gap between the HELENA battery chemistry and existing industrial recycling methods. The goal is to harmonize the developed materials with established recycling infrastructures, ensuring they can be efficiently processed within current industrial frameworks rather than requiring entirely new, cost-intensive solutions.

HELENA’s proposed recycling pathways will be tested through experimental evaluation. Data gathered from these recycling processes, alongside insights from battery manufacturing, will inform a comprehensive life cycle assessment (LCA). This assessment will provide a comprehensive evaluation of environmental and social impacts, guiding strategies for mitigation, elimination, or compensation of adverse effects. Additionally, emissions monitoring at both the source and receiver levels will ensure that sustainability measures are effectively implemented. The results will inform practical sustainability strategies, driving long-term improvements and supporting structured implementation across the entire recycling process.

## 4 Conclusions

HELENA is a collaborative project aiming to develop the next generation of Li-metal halide-based SSBs, with specific application for electric vehicles and aircrafts. By integrating a high-voltage Ni-rich layered oxide cathode, a high-energy Li metal anode, and a Li-ion superionic halide solid electrolyte, HELENA aims to address critical limitations in conventional lithium-ion batteries, notably in terms of energy density, power performance, safety, and cost-effectiveness. These cells target to reach energy densities up to 400 Wh kg^–1^ and 1200 Wh L^–1^, positioning them as competitive alternatives to current Li-ion technologies.

The project has successfully demonstrated key innovations in the development of halide electrolytes, with element doping strategies improving ionic conductivity up to 5.1 mS cm^–1^. Additionally, the project is accomplishing the optimization of the methods for processing materials into battery components for their integration into pre-industrial prototype cells. The adoption of a multiscale-multiphysics modelling has allowed to optimize battery architecture and performance, accounting for phenomena across atomic, microscale, and macroscale levels. This modelling framework has been critical in understanding the interactions between materials, predicting ionic transport, mechanical stresses, and mitigating issues like dendrite formation, which are crucial for the long-term stability and safety of lithium metal-based SSBs. Moreover, the project’s focus on sustainability has led to the development of robust recycling strategies with high material recovery rates tailored to the unique challenges of halide-based SSBs.

Building on the promising results from this project, future research will focus on further scaling up of the technology. We expect that the manufactured devices will help to push the boundaries of energy density, safety and sustainability, paving the way for next-generation batteries that meet the increasing demands of modern society, while minimizing environmental impact.

## CRediT authorship contribution statement

**Fathi Reza:** Writing – review & editing, Validation, Resources, Formal analysis. **Hatz Anna-Katharina:** Writing – review & editing, Investigation. **Marchandier Thomas:** Writing – review & editing, Validation, Resources, Investigation, Formal analysis. **Tron Artur:** Writing – review & editing, Validation, Investigation, Formal analysis. **Gavagnin Claudio:** Writing – review & editing, Validation, Investigation, Formal analysis. **Varkey Cerun Alex:** Writing – review & editing, Validation, Software, Methodology, Investigation, Formal analysis. **Chaykina Diana:** Writing – review & editing, Validation, Resources, Investigation, Formal analysis. **Xu Qi:** Writing – review & editing, Validation, Resources, Investigation, Formal analysis. **Gonzalo Elena:** Validation, Investigation, Formal analysis. **Golov Andrey:** Writing – review & editing, Visualization, Validation, Investigation, Formal analysis. **Carrasco Javier:** Writing – review & editing, Validation. **Abada Sara:** Writing – review & editing, Validation, Software, Methodology, Investigation, Formal analysis. **Drude Emanuel:** Writing – review & editing, Validation, Investigation, Formal analysis. **Diaz Fabian:** Writing – review & editing, Validation, Investigation, Formal analysis. **Hamzelui Niloofar:** Validation, Investigation, Formal analysis. **Robles-Fernandez Adrian:** Writing – review & editing, Visualization, Validation, Investigation, Formal analysis. **Brunetti Massimo:** Validation, Formal analysis. **Lannelongue Pierre:** Writing – review & editing, Visualization, Validation, Investigation, Formal analysis. **Sudharshan Akshayan:** Writing – review & editing, Validation, Investigation, Formal analysis. **López-Aranguren Pedro:** Writing – original draft, Visualization, Supervision, Project administration, Funding acquisition, Formal analysis, Conceptualization. **Guy Nicolas:** Validation, Investigation, Formal analysis. **Beschnitt Stefan:** Writing – review & editing, Validation. **Eshetu Gebrekidan Gebresilassie:** Validation, Investigation, Formal analysis. **Almousli Alaa:** Validation, Formal analysis. **Kaminski Matteo:** Writing – review & editing, Validation, Investigation, Formal analysis.

## Declaration of Competing Interest

The authors declare that they have no known competing financial interests or personal relationships that could have appeared to influence the work reported in this paper.
