# Supplementary material for: HELENA project: Driving innovation in high energy density Li-metal halide solid-state batteries for electric vehicles and aircrafts
Source: Comput Struct Biotechnol J. 2025 Mar 19;29:72–84. doi: 10.1016/j.csbj.2025.03.013 (PMC11985150; doi:10.1016/j.csbj.2025.03.013)
Supplement: Supplementary file 1 — Supplementary material [file mmc1.docx]

**SUPPORTING INFORMATION FOR:**

**HELENA project: Driving innovation in high energy density Li-metal halide solid-state batteries for electric vehicle and aircraft**

**Table 1.** Comparison of conventional LiBs, oxide-, sulfide- and halide-based solid-state batteries.

| **Battery technology** | **Cathode-Anode Chemistry** | **Electrolyte** | **Wh/kg** | **Cycle life (cycles)** | **Cost** | **Refs.** |
| --- | --- | --- | --- | --- | --- | --- |
| LiBs | NMC-Graphite / LFP-Graphite | Liquid organic electrolyte | 250-300 | >2000 | $40-140/kWh | [1–3] |
| SSB  Oxide-based | NMC,LCO\|Oxide,Phosphate\|LiM | Oxide (i.e.,LLZO, LATP) | 320 | >1000 | $70/kWh | [4–6] |
| SSB  Sulfide-based | NMC,LCO\| Sulfide\|LiM | Sulfide (i.e.,LPS,LPSCl,LGPS) | 400-450 | >1000 | >$100/kWh | [7–11] |
| SSB  Halide-based | NMC, LCO\|Halide\|LiM | Halide (i.e.,Li3InCl6​, Li3​YCl6​) | 400-500 | >1000 | Target <$75/kWh | [12,13] |

In Figure S1, thermochemical simulations using FactSage^TM^ 8.0 Database PS are presented. These simulations assess the lithium's behaviour in the presence of nitrogen, oxygen, and water (H_2_O) during heating. The colour variations in the figures represent the enthalpy values. This provides insights into the risks associated with excess energy release in straightforward reactions, resulting in the formation of main products like Li_3_N, Li_2_O, and LiH. The dotted lines in the figures represent the portion of metallic lithium that transforms into the target compound. It is important to note that the simulations were conducted using 100 g of metallic lithium with a variable addition of the interacting media during heating.

From Figure S1, the following can be highlighted:

Figure S1A: Thermal conditioning under nitrogen leads to direct formation of Li_3_N with a mild possibility of heat generation compared to results using oxygen or water.

Figure S1B: Conditioning with oxygen leads to the most exothermic reaction compared to other media. This indicates that thermal treatment of this type of material will result in the formation of Li_2_O.

Figure S1C and Figure 8D: Indicate two species formed if lithium is heated in the presence of water. These resulting stable species are Li_2_O and LiH. It is worth highlighting that Li_2_O remains the primary energy source due to its high exothermic characteristics. Lithium is also more likely to form this oxide than any other. The simulation intends only to indicate the reaction mechanism and not the final product. In this term, metastable compounds might differ from final products.

Figure 1: Behaviour of metallic lithium during thermal conditioning in the presence of A) Formation of Li_2_O in nitrogen, B) Formation of Li_2_O in Oxygen, C) Formation of Li_2_O in Water (H_2_O), and D) Formation of LiH in Water (H_2_O)

During thermal treatment, introducing other gases can lead to more complex reactions and the formation of multi-phase systems. The inclusion of CO_2_ exemplifies this. This gas, commonly used in various thermal treatment applications, introduces two elements, carbon and oxygen, to lithium. Figure 9 illustrates that stable Li_2_O and Li_2_C_2_ are formed in this scenario. It is noticeable that as the concentration of CO_2_ increases in the system, lithium transforms into both species. However, if this gas is in excess, Li_2_O tends to dominate. Interestingly, this combination appears unaffected by temperature variations. It is worth noting that these simulations are purely used as indication and not as absolute prediction of the phenomena. The software used for simulation cannot simulate the kinetics of the reaction. Therefore, some metastable phases may be formed but not shown in the results. As an example, it is Li_2_CO_3_. Only real experimental data could give a closer view of the phenomena and species being formed.

Enthalpy change (ΔH) serves as a measure of the heat energy transferred during a process, spanning from the initial reactants to the final products. This parameter aids in categorizing reactions as either exothermic (resulting in heat release, ΔH < 0) or endothermic (resulting in heat absorption, ΔH > 0). In this context, the enthalpy changes during the process indicate Li_2_O as the most exothermic reaction in this transition, making it the most likely outcome.


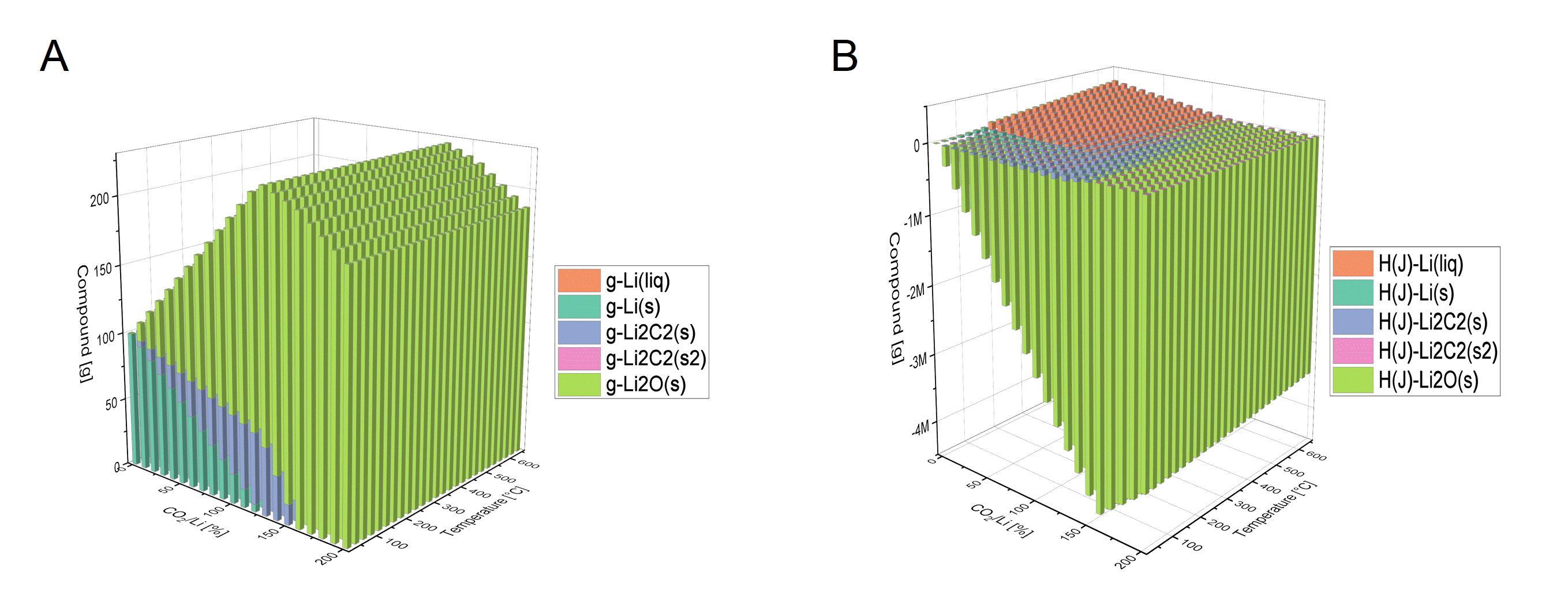


Figure S2: Thermochemical interaction between metallic lithium and CO_2_ during thermal treatment: A) Compound formation, B) Resulting enthalpy of main species

The possibility of a two-stage reaction in this kind of thermal treatment is highly likely. Hence, it is crucial to understand the thermochemical stability of the reported compounds, such as Li_2_O and Li_3_N. For these two species, it is essential to establish their stability in the presence of water, which can be in liquid or gas form. The resulting thermochemical modeling is presented in Figure S3 and Figure S4 for Li_2_O and Li_3_N, respectively.

Figure S3: Thermochemical interaction between Li_2_O and H_2_O during thermal treatment: A) Compound formation, B) Resulting enthalpy of main species

Based on the figures, it is imperative to note that Li_3_N rapidly transforms into LiOH (in solid or liquid form), Li_2_O, and nitrogen. Of particular concern is the presence of NH_3_ in gas form at temperatures below 200 °C. This could potentially lead to safety issues, as NH_3_ is known for its environmental hazards if not handled properly. With further heating, the NH_3_ molecule splits into nitrogen and free hydrogen. As indicated in the enthalpy data, it is again evident that Li_2_O remains the primary exothermic reaction in the system, followed by LiOH formation.

Figure S4: Thermochemical interaction between Li_3_N and H_2_O during thermal treatment: A) Compound formation, B) Resulting enthalpy of main species

**References**

[1] N. Nitta, F. Wu, J.T. Lee, G. Yushin, Li-ion battery materials: present and future, Materials Today 18 (2015) 252–264. https://doi.org/10.1016/j.mattod.2014.10.040.

[2] H. Niu, N. Zhang, Y. Lu, Z. Zhang, M. Li, J. Liu, N. Zhang, W. Song, Y. Zhao, Z. Miao, Strategies toward the development of high-energy-density lithium batteries, Journal of Energy Storage 88 (2024) 111666. https://doi.org/10.1016/j.est.2024.111666.

[3] Fast charging of energy-dense lithium-ion batteries | Nature, (n.d.). https://www.nature.com/articles/s41586-022-05281-0 (accessed March 5, 2025).

[4] Solid-State Battery Roadmap 2035+, n.d. https://www.isi.fraunhofer.de/content/dam/isi/dokumente/cct/2022/SSB_Roadmap.pdf.

[5] Quantum Scape 2021 Investor Presentation 2021: August 2021 https://s26.q4cdn.com/263384136/files/doc_presentation/ 2021/08/Investor-Presentation_Sep_2021.pdf (accessed 21 Dec 2021), n.d.

[6] Ampcera 2021 Technology: Highlights of Ampcera’s solid-state electrolyte (SSE) technologies https://ampcera.com/ index.php/technology/, n.d.

[7] L. Liu, J. Xu, S. Wang, F. Wu, H. Li, L. Chen, Practical evaluation of energy densities for sulfide solid-state batteries, eTransportation 1 (2019) 100010. https://doi.org/10.1016/j.etran.2019.100010.

[8] A. Joshi, D.K. Mishra, R. Singh, J. Zhang, Y. Ding, A comprehensive review of solid-state batteries, Applied Energy 386 (2025) 125546. https://doi.org/10.1016/j.apenergy.2025.125546.

[9] S. Randau, D.A. Weber, O. Kötz, R. Koerver, P. Braun, A. Weber, E. Ivers-Tiffée, T. Adermann, J. Kulisch, W.G. Zeier, F.H. Richter, J. Janek, Benchmarking the performance of all-solid-state lithium batteries, Nature Energy 5 (2020) 259–270. https://doi.org/10.1038/s41560-020-0565-1.

[10] Y.-K. Sun, Emerging All-Solid-State Lithium–Sulfur Batteries: Holy Grails for Future Secondary Batteries, ACS Energy Lett. 9 (2024) 5092–5095. https://doi.org/10.1021/acsenergylett.4c02563.

[11] W. Zaman, K.B. Hatzell, Processing and manufacturing of next generation lithium-based all solid-state batteries, Current Opinion in Solid State and Materials Science 26 (2022) 101003. https://doi.org/10.1016/j.cossms.2022.101003.

[12] C. Wang, J. Liang, J.T. Kim, X. Sun, Prospects of halide-based all-solid-state batteries: From material design to practical application, Science Advances 8 (2022) eadc9516. https://doi.org/10.1126/sciadv.adc9516.

[13] Halide solid-state electrolytes for all-solid-state batteries: structural design, synthesis, environmental stability, interface optimization and challenges - Chemical Science (RSC Publishing), (n.d.). https://pubs.rsc.org/en/content/articlelanding/2023/sc/d3sc02093b (accessed November 25, 2024).
